# Down-Regulation of Small Rubber Particle Protein Expression Affects Integrity of Rubber Particles and Rubber Content in *Taraxacum brevicorniculatum*

**DOI:** 10.1371/journal.pone.0041874

**Published:** 2012-07-23

**Authors:** Andrea Hillebrand, Janina J. Post, David Wurbs, Daniela Wahler, Malte Lenders, Vladislav Krzyzanek, Dirk Prüfer, Christian Schulze Gronover

**Affiliations:** 1 Institute of Plant Biology and Biotechnology, Westphalian Wilhelms-University, Münster, Germany; 2 Institute for Medical Physics and Biophysics, Westphalian Wilhelms-University, Münster, Germany; 3 Fraunhofer Institute for Molecular Biology and Applied Ecology, Aachen, Germany; 4 Institute of Scientific Instruments of the ASCR, v.v.i., Academy of Sciences of the Czech Republic, Brno, Czech Republic; Centro de Investigación y de Estudios Avanzados del IPN, Mexico

## Abstract

The biosynthesis of rubber is thought to take place on the surface of rubber particles in laticifers, highly specialized cells that are present in more than 40 plant families. The small rubber particle protein (SRPP) has been supposed to be involved in rubber biosynthesis, and recently five SRPPs (TbSRPP1–5) were identified in the rubber-producing dandelion species *Taraxacum brevicorniculatum*. Here, we demonstrate by immunogold labeling that TbSRPPs are localized to rubber particles, and that rubber particles mainly consist of TbSRPP3, 4 and 5 as shown by high-resolution two-dimensional gel electrophoresis and mass spectrometric analysis. We also carried out an RNA-interference approach in transgenic plants to address the function of TbSRPPs in rubber biosynthesis as well as rubber particle morphology and stability. TbSRPP-RNAi transgenic *T. brevicorniculatum* plants showed a 40–50% reduction in the dry rubber content, but neither the rubber weight average molecular mass nor the polydispersity of the rubber were affected. Although no phenotypical differences to wild-type particles could be observed *in vivo*, rubber particles from the TbSRPP-RNAi transgenic lines were less stable and tend to rapidly aggregate in expelling latex after wounding of laticifers. Our results prove that TbSRPPs are very crucial for rubber production in *T. brevicorniculatum*, probably by contributing to a most favourable and stable rubber particle architecture for efficient rubber biosynthesis and eventually storage.

## Introduction

Approximately 2,500 plants produce natural rubber poly (*cis*-1,4-isoprene), which is stored in rubber particles dispersed throughout the cytosol of either laticifers or specialized parenchyma cells [1]. Natural rubber is synthesized by the successive addition of isopentenyl pyrophosphate (IPP) to an initiating allylic pyrophosphate, e.g. farnesyl pyrophosphate (FPP) [1]–[5]. This polymerization reaction is thought to take place on the surface of rubber particles, mediated by a particle bound *cis*-prenyltransferase (CPT) [1], [6]–[8]. Recently, we identified three rubber particle bound long-chain CPTs in *Taraxacum brevicorniculatum* (*T. brevicorniculatum* was erroneously labeled as *T. koksaghyz* in botanical gardens [9]) that appear to be the key enzymes for rubber biosynthesis in this species [8], [10]. Detailed analysis of rubber particles in different species revealed a conserved spherical structure with sizes ranging from 0.2–6.5 µm in *Ficus carica* and *Ficus benghalensis*, 1–2 µm in *Parthenium argentatum,* 0.08–2 µm in *Hevea brasiliensis* and 0.01–1 µm in *T. brevicorniculatum* [8], [11]–[13]. Rubber particles comprise a homogenous rubber core surrounded by a monolayer membrane containing lipids, proteins, and several minor compounds [12], [14], [15]. This composite monolayer membrane probably mediates the colloidal stability of rubber particles by providing a net negative surface charge that prevents their coalescence in the cytosol [12], [14]. In principle, colloidal stability depends on the balance between repulsion and attractive van-der-Waals forces, and electrostatic forces are often responsible for the repulsion of colloids in a suspension [16]–[18]. The net surface charge of colloids can be estimated by the determination of their zeta (ζ)-potential, which is the electrical potential difference at the interface of the suspending fluid and the stationary fluid attached to the colloid surface [16], [18]. In addition to repulsion through a similar (positive or negative) surface charge, repulsion of colloids can also be mediated by steric hindrance of adsorbed large molecules, such as proteins [17], [19]–[22]. Amongst others, two highly homologous proteins are associated with the surface of *H. brasiliensis* rubber particles, i.e. the small rubber particle protein (SRPP; here designated as HbSRPP) and the rubber elongation factor (REF; here designated as HbREF) [23]–[25]. SRPP homologs were also identified in *P. argentatum* (guayule homolog of SRPP, GHS [26]) and *T. brevicorniculatum* (TbSRPP1–5, previously designated as TkSRPP1–5 [27]). The functions of SRPP and REF are yet unclear. However, there is some evidence indicating a potential role in rubber biosynthesis: *In vitro*-experiments revealed that the addition of recombinant HbSRPP to *H. brasiliensis* rubber particles suspended in latex cytosol increased the incorporation of IPP, while addition of antibodies directed against HbSRPP and HbREF inhibited IPP incorporation [24], [25]. Moreover, the analysis of HbREF gene expression in *H. brasiliensis* accessions with low to high rubber yields revealed that HbREF expression correlates positively with the amount of rubber [28]. However, it has been proposed that the rubber-producing species *F. carica* and *F. benghalensis* do not possess a SRPP homolog, suggesting that SRPP is not a general and essential protein for rubber biosynthesis [13].

The recently identified SRPPs in *T. brevicorniculatum* (TbSRPP1–5) shared sequence identities of 41–66% to each other and 38–43% with HbSRPP. Except for TbSRPP2, all TbSRPPs are predominately expressed in latex and their spatiotemporal expression pattern correlates with latex and rubber production in *T. brevicorniculatum* [27]. To analyze the function of TbSRPPs *in vivo*, we generated transgenic *T. brevicorniculatum* plants with strongly reduced TbSRPP protein levels, caused by RNA-interference. We revealed that exuded latex as well as freeze-dried roots of transgenic plants contain significantly lower amounts of natural rubber compared to wild-type plants. Rubber particles in expelling latex of transgenic plants appeared to be less stable compared to wild-type, whereas neither the rubber weight average molecular mass nor the polydispersity were affected. Hence, we conclude that TbSRPPs probably support rubber biosynthesis by the stabilization of rubber particles.

## Results

### TbSRPPs are the major rubber particle proteins

**Figure 1:**
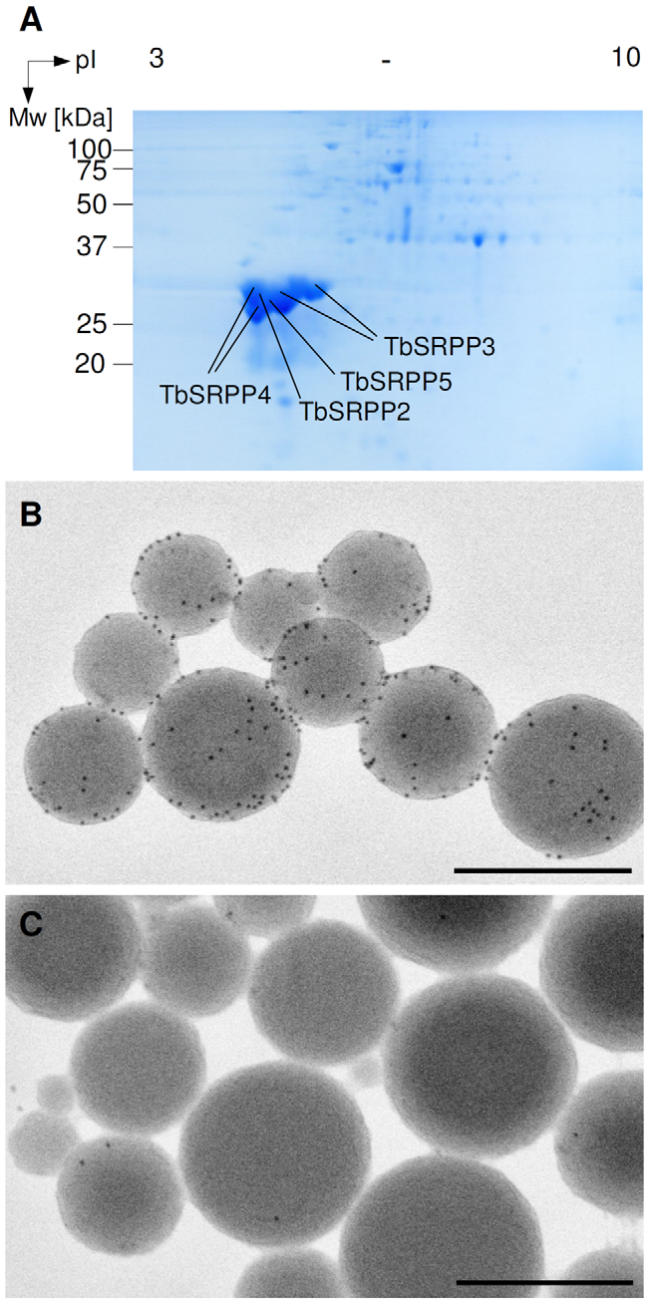
Association of TbSRPPs with rubber particles. A. Fifty micrograms of purified rubber particle proteins were separated by isoelectric focussing (pH 3–10 IPG strips) and subsequent SDS-PAGE. The gel was stained with colloidal Coomassie Brilliant Blue. **B.** Backscattered electron imaging of rubber particles labeled with 10 nm gold particles by immunodetection using the TbSRPP-antibody. **C.** Gold labeling of rubber particles was not appreciably detected in backscattered electron images, when the corresponding pre-immune serum was used as primary antibody. Micrographs are shown as inverted images. Scale bars  = 400 nm.

We recently identified and cloned five genes (*TbSRPP1–5*) encoding small rubber particle proteins from *T. brevicorniculatum* [27]. As reported for other SRPPs [13], [25], we first set out to determine whether TbSRPP1–5 are associated with rubber particles by two-dimensional (2-D) gel electrophoresis followed by mass spectrometry. We revealed a cluster of highly abundant protein spots with isoelectric point (pI) values between pH 4 and pH 6 (Fig. 1A). Mass spectrometric (MS) analysis of these protein spots generated representative peptide sequences for TbSRPP2, TbSRPP3, TbSRPP4 and TbSRPP5, whereas no peptides were detected for TbSRPP1 (Tab. S1). The most abundant rubber particle protein was TbSRPP3, followed by TbSRPP4 and TbSRPP5, while TbSRPP2 was present only in small amounts (Fig. 1A, Tab. S1).

Next, the presence of TbSRPPs on rubber particles was investigated by scanning electron microscopy (SEM) using a polyclonal antibody raised against TbSRPP3. In Western blot experiments, this antibody recognized all TbSRPPs with the same efficiency, most probably due to the high amino acid sequence conservation between members of this protein family [27], and will therefore be referred to as anti-TbSRPP (Fig. S1, Text S1). Immunogold labeling of rubber particles using anti-TbSRPP and a gold-coupled secondary antibody with subsequent visualization by SEM directly confirmed the presence of TbSRPPs on the rubber particle surface (Fig. 1B). Control experiments with pre-immune serum did not show significant staining of rubber particles (Fig. 1C).

### Depletion of TbSRPP1-5 by RNA-interference

**Figure 2:**
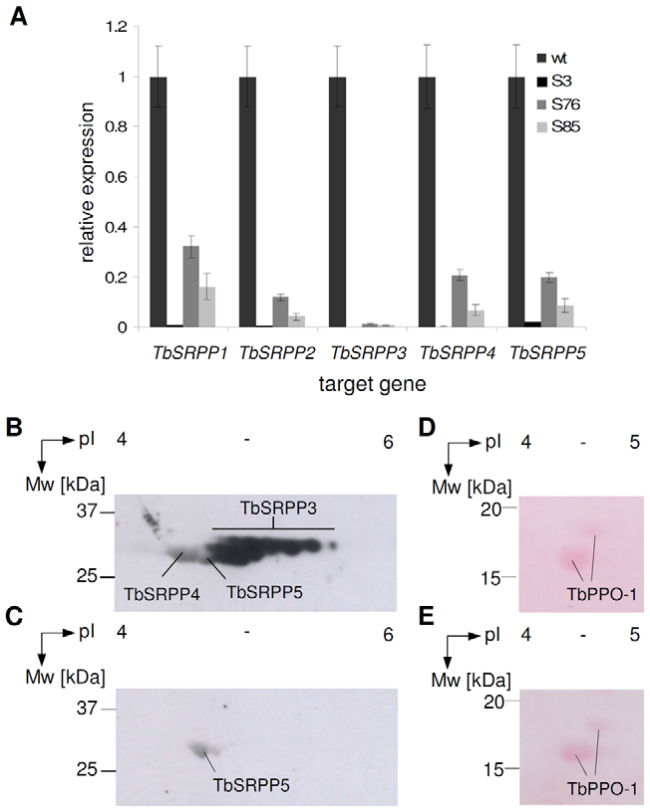
Analysis of *TbSRPP1–5* expression in TbSRPP-RNAi transgenic and wild-type plants. A. *TbSRPP* transcription levels in the latex of three independent TbSRPP-RNAi transgenic lines (designated S3, S76 and S85) were quantified by RT-PCR. Relative transcription of *TbSRPPs* was normalized to the constitutively expressed control gene *TbACTIN*. The ratios of *TbSRPPs*/*TbACTIN* transcription levels in wild-type (wt) served as a reference and were set to the value of one. Values represent mean ± SE (*n* = 3). **B, C.** Fifty microgram of rubber particle proteins from wild-type (B) and TbSRPP-RNAi lines (C, exemplarily shown for line S3) was separated by isoelectric focussing (pH 3–6 IPG strips) and subsequent SDS-PAGE. Proteins were transferred to nitrocellulose membranes and TbSRPPs were detected using an anti-TbSRPP antibody and a secondary antibody conjugated with horse radish peroxidase. **D, E.** Ponceau-S staining of the nitrocellulose membranes confirmed equal loading of gels prepared for wild-type (D) and line S3 (E), by showing that proteins spots of the C-terminus of the Polyphenoloxidase-1 (TbPPO-1) were present in similar amounts [29], [30].

To further address the function of TbSRPPs in rubber biosynthesis we developed transgenic *T. brevicorniculatum* plants with reduced *TbSRPPs* expression levels by RNA-interference (RNAi). Therefore, we introduced a *TbSRPP3*-RNAi construct into the genome of *T. brevicorniculatum* by *Agrobacterium-*mediated transformation. Out of ten independently regenerated transgenic plants, six lines displayed reduced transcript levels for all *TbSRPP* genes and were therefore designated as TbSRPP-RNAi transgenic plants (data not shown). Subsequently, three TbSRPP-RNAi transgenic plants, labeled S3, S76 and S85, were selected for further analysis of *TbSRPP1–5* expression by quantitative RT-PCR using total RNA from latex. The expression of all *TbSRPP* genes was severely reduced by the *TbSRPP3*-RNAi construct when compared to wild-type plants (Fig. 2A). The most severe reduction in *TbSRPPs* expression was found in S3, where only residual amounts of *TbSRPP5* are still visible.

Next, the effect of the *TbSRPP3*-RNAi construct was analyzed on protein level by 2-D gel electrophoresis of rubber particle proteins followed by immunodetection using the anti-TbSRPP antibody. Equal loading of the gels was verified after Ponceau-S staining of the nitrocellulose membrane by comparing the intensities of the protein spots representing the C-terminus of the Polyphenoloxidase 1 (TbPPO-1), which is expressed consistently in *T. brevicorniculatum* and thus suitable as loading control [29], [30]. Compared to wild-type plants (Fig. 2B), the levels for TbSRPPs were strongly reduced as exemplarily shown for S3 (Fig. 2C). It should be noted that *TbSRPP* transcription (Fig. 2A) strongly correlates with the observed protein levels as only residual amounts were identified for TbSRPP5 by 2-D gel electrophoresis (Fig. 2C). The transgenic lines showed regular growth and development under standard greenhouse conditions.

### Reduced TbSRPP protein levels had severe impacts on rubber production

To test if depleted TbSRPP levels affect rubber biosynthesis, the dry rubber content in exuding latex and freeze-dried roots was measured. Compared to wild-type, the latex of TbSRPP-RNAi transgenic plants contained significantly lower amounts of rubber (24–37% of wild-type, Tab. 1) and the same was evident for the rubber content of freeze-dried roots (Tab. 1). Additionally, a clear correlation between *TbSRPP* expression levels and rubber content was indicated (Tab. 1, see also Fig. 2A).

**Table 1:** Table **1.** Content, molecular mass and polydispersity of rubber from exuded latex and freeze-dried roots and corresponding long-chain prenyltransferase activity.

plant lines	*latex dry rubber content*	*root dry rubber content*	*rubber molecular mass distribution* a			*[1-^14^C] IPP transferase activty*
	[mg ml^−1^]	[mg ml^−1^]	Μw^b^ [10^6^ Da]	Μn^c^ [10^6^ Da]	ΜwΜn^−1^	[pmol mg^−1^ min^−1^]
wt	172.6 (±11.3)	3.88 (±0.65)	3.30 (±0.47)	2.21 (±0.32)	1.56 (±0.03)	1.83 (±0.29)
S3	41.5 (±8.3) **	1.93 (±0.17)**	2.74 (±0.23)	1.97 (±0.15)	1.39 (±0.04)	1.09 (±0.04)*
S76	63.2 (±8.6) **	2.32 (±0.09)**	2.69 (±0.84)	1.86 (±0.11)	1.45 (±0.04)	0.96 (±0.09)**
S85	61.4 (±0.4) **	2.15 (±0.12)**	3.14 (±0.27)	1.99 (±0.34)	1.62 (±0.23)	1.29 (±0.20)*

The values are given as the mean of at least three repititions ± SD.

a Molecular mass distribution was measured by size exclusion chromatography of rubber from exuded latex.

b W, weight average molecular mass.

c N, number average molecular mass.

* statistically significant difference compared to wild type control (P, 0.05).

** statistically significant difference compared to wild type control (P, 0.01).

Next, we analyzed the weight average molecular mass (Μw) and number average molecular mass (Μn) of rubber obtained from exuding latex. Using size exclusion chromatography (SEC) no significant differences could be detected between rubber of wild-type and TbSRPP-RNAi transgenic plants indicating that polymer length and polydispersity of the residual rubber were not influenced by altered TbSRPPs expression (Tab. 1).

To figure out whether reduced TbSRPP levels affected the activity of the long chain prenyltransferase in latex, we carried out *in vitro* [1-^14^C] IPP incorporation assays using whole latex from TbSRPP-RNAi transgenic and wild-type plants as described by Post *et* *al*. [10]. After removal of the short- and medium-chain polyprenol diphosphates by *n*-butanol extraction, the long-chain products were obtained with a mixture of toluene and hexane and the incorporated radioactivity was measured. This experiment revealed that the activity of the long-chain prenyltransferase in latex of TbSRPP-RNAi transgenic plants was indeed significantly lower under this condition (Tab. 1).

### Silencing of TbSRPPs reduces the stability of rubber particles in exuding latex

**Figure 3:**
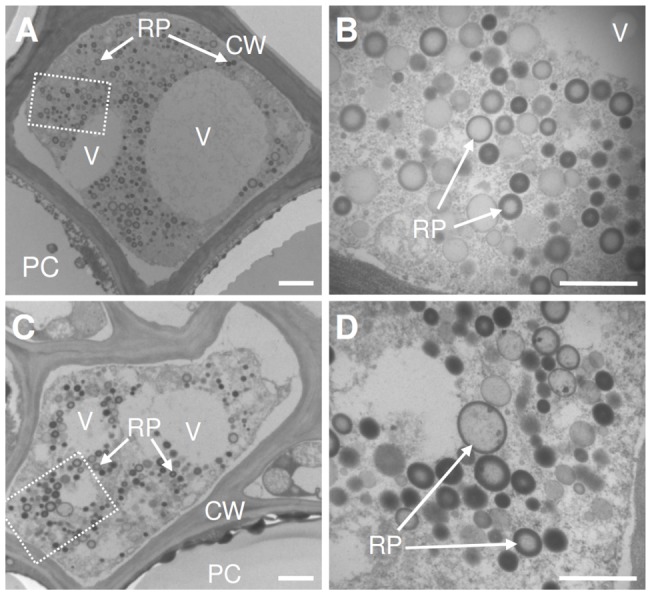
*In situ* analysis of the ultra-structure of rubber particles from TbSRPP-RNAi transgenic plants and wild-type plants by transmission electron microscopy. **A.** Micrograph of ultra-thin cross sections of roots from TbSRPP-RNAi transgenic plant (S3) at low and **B.** higher magnification. **C.** Micrograph of ultra-thin cross sections of roots from a wild-type plant at low and **D.** higher magnification. Enlarged areas are indicated with dashed boxes. Key: RP, rubber particle; V, vacuole; CW, cell wall; PC, parenchyma cell. Scale bars  = 1 µm.

We set out to analyze if the depleted levels of TbSRPPs affected the architecture of rubber particles by ultrastructural investigations using transmission electron microscopy (TEM). TEM images of ultra-thin cross sections from roots of TbSRPP-RNAi transgenic (Fig. 3A) and wild-type plants (Fig. 3C) showed laticifers with electron dense material, organelle-like structures, and rubber particles. In the analyzed sections, rubber particles appeared spherical and did not show any obvious morphological differences in both, TbSRPP-RNAi transgenic (Fig. 3B) and wild-type (Fig. 3D) plants.

**Figure 4:**
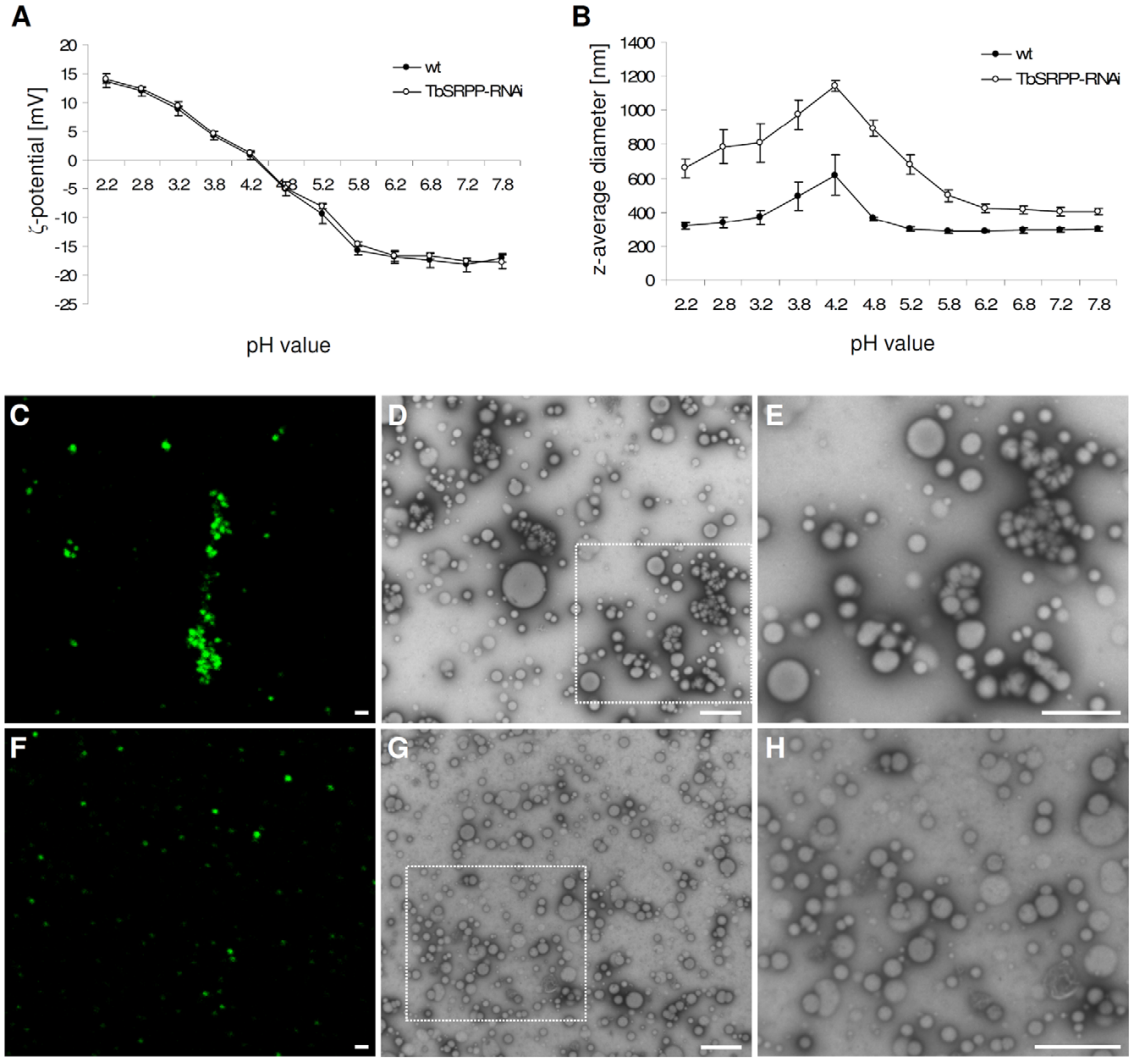
*In vitro* analysis of the colloidal stability of rubber particles from TbSRPP-RNAi transgenic plants and wild-type plants. **A.** Measurement of the zeta (ζ)-potential of rubber particles from wild-type plants (filled circles) and TbSRPP-RNAi transgenic plants (open circles) suspended in modified rubber extraction buffer (REB) at given pH values. Values represent mean ± SD of three independent plant lines (S3, S76 and S85) respectively of six wild-type plants (wt). No significant differences were detected among the plants using Students t-test (P, 0.05). **B.** Measurement of the z-average diameter of rubber particles from wild-type plants (filled circles) and TbSRPP-RNAi transgenic plants (open circles) suspended in modified REB with the indicated pH values. Values represent mean ± SD of three independent plant lines (S3, S76 and S85) respectively of six wild-type plants (wt). Significant differences have been detected among TbSRPP-RNAi lines and wild-type plants using Students t-test (P, 0.01). **C, F.** Confocal laser scanning microscopic analysis of isolated rubber particles from TbSRPP-RNAi transgenic plants (C) and wild-type plants (F) that were stained with nile red. **D, E** **and** **G, H.** Transmission electron microscopic analysis of isolated rubber particles from TbSRPP-RNAi transgenic and wild-type plants. **D.** Micrograph of isolated rubber particles from TbSRPP-RNAi transgenic plant line S3 suspended in modified rubber extraction buffer (REB) pH 7.2 at low and **E.** higher magnification. **G.** Micrograph of rubber particles from wild-type suspended in modified REB pH 7.2 at low and **H.** higher magnification. Enlarged areas are indicated with dashed boxes. Scale bars = 1 µm.

We further studied the colloidal stability of purified rubber particles from exuding latex of TbSRPP-RNAi transgenic and wild-type plants by measuring their ζ-potential at different pH values. Both types of rubber particles showed a similar ζ-potential ranging from +14 mV at pH 2.2 to −18 mV at pH 7.2 (Fig. 4A). The pI was approximately pH 4.2. In contrast to the ζ-potential, significant differences were obtained for their size. Rubber particles from wild-type latex revealed a z-average size of 295 nm at pH 7.2 (Fig. 4B) and showed an increased diameter at pH-values near their pI (pH 3.2–4.8, Fig. 4B). Rubber particles obtained from TbSRPP-RNAi transgenic plants, however, displayed with 406 nm at pH 7.2 a significantly higher z-average diameter compared to the wild-type control. A similar increase in z-average diameter was also evident at other pH values (Fig. 4B).

The increase of the z-average diameter could indicate an aggregation of rubber particles, although such structures have not been observed *in situ* (Fig. 3). To proof if the differences in z-average diameter point toward the formation of rubber particle aggregates, we stained the rubber particles with the lipophilic fluorescent dye nile red [31] and analyzed the samples by confocal laser scanning microscopy (CLSM, Fig. 4C, F). While the rubber particles of TbSRPP-RNAi transgenic plants formed large aggregates at pH 7.2, their wild-type counterparts appeared to be evenly distributed in the suspending buffer. For more detailed observations we again carried out TEM-analysis and confirmed that rubber particles fromTbSRPP-RNAi transgenic plants indeed tend to aggregate at pH 7.2 and in some cases are also partially fused (Fig. 4D, E). No similar observations were made for rubber particles of wild-type latex (Fig. 4G, H).

## Discussion

The biosynthesis of rubber at the surface of rubber particles is only little understood. Recently, CPTs were identified as one important component of the polymer elongation process in *T. brevicorniculatum* [10], however other proteinaceous and non-proteinaceous factors appear to be necessary for efficient rubber production.

In this study we showed that the three SRPP homologs TbSRPP3, 4 and 5 are the major rubber particle proteins in *T. brevicorniculatum* and confirmed their association with rubber particles by immunogold labeling and SEM (Fig. 1, Tab. S1). While we were also able to detect small amounts of TbSRPP2 at the rubber particle surface, TbSRPP1 seems to be not or only in non-detectable amounts (by 2D-gel electrophoresis) associated with the rubber particle surface, since no peptide sequences for TbSRPP1 could be detected. This could result from the washing steps during the purification procedure applied to the rubber particles by which more loosely associated proteins might detach from the rubber particle surface (Fig. 1A, Tab. S1). In *H. brasiliensis,* the closely related proteins HbREF and HbSRPP are also associated with rubber particles, whereby HbREF is the most abundant rubber particle protein followed by HbSRPP [13], [24], [25]. HbREF and HbSRPP as well as their homolog GHS from *P. argentatum* have been supposed to positively influence rubber biosynthesis: It has been shown that heterologously expressed GST-HbSRPP as well as His-GHS fusion proteins enhance IPP incorporation activity of rubber particles *in vitro*, while immunoinhibition by appropriate HbREF and HbSRPP antibodies reduces the IPP incorporation [24]–[26]. Therefore, it has been suggested that HbSRPP is part of the rubber transferase complex or that it is involved in the synthesis or recruitment of substrates for rubber biosynthesis initiation and/or rubber chain elongation [25]. However, Singh and co-workers speculated that SRPP is not crucial for rubber biosynthesis since its detection on *F. carica* and *F. benghalensis* rubber particles by immunogold labeling with an HbSRPP antibody failed [13]. As SRPP homologs have been detected in species that produce rubber of high molecular mass (*H. brasiliensis* and *P. argentatum*) but not in species possessing only low molecular mass rubber (*F. carica* and *F. benghalensis*), it has been supposed that HbSRPP and GHS might be important factors for determination of the rubber molecular mass by assisting CPT function [26]. Due to the lack of *in vivo* evidence, the roles of SRPP, REF, and GHS in rubber biosynthesis have yet not been sufficiently proven.

To elucidate the role of TbSRPPs in rubber biosynthesis *in vivo*, we generated transgenic plants expressing an RNAi construct directed against *TbSRPPs*. Quantitative RT-PCR and 2-D gel electrophoresis with subsequent immunodetection using the anti-TbSRPP antibody confirmed that expression of all TbSRPPs was severely silenced in the transgenic lines (Fig. 2). By determination of the dry rubber content from exuded latex and freeze-dried roots we were able to show that the TbSRPP-RNAi transgenic plants contained significantly lower amounts of natural rubber compared to wild-type controls (Tab. 1) indicating an impaired rubber biosynthesis in the TbSRPP-RNAi transgenic lines. In contrast, SEC-analysis of the rubber samples revealed a wild-type like rubber chain elongation since neither the weight average molecular mass nor the polydispersity was affected by reduced TbSRPP protein levels (Tab. 1). Therefore, we propose that TbSRPPs are not mandatory for the formation of high molecular mass rubber. Instead, the rubber biosynthesis seems to be less effective in TbSRPP-RNAi transgenic plants, probably resulting from an inefficient enzymatic activity of the long-chain prenyltransferase (TbCPT1–3) [10], as also suggested by the data obtained from the *in vitro* prenyltransferase assay (Tab. 1).

Rubber particles are not only required for the storage of rubber molecules, but also to provide a suitable and stable platform for rubber biosynthesis. Although one could expect that the depletion of major integral rubber particle proteins might have a dramatic effect on the morphology of rubber particles, TEM-analysis of ultra-thin cross sections of laticifers revealed no obvious differences in the architecture of rubber particles from TbSRPP-RNAi transgenic and wild-type plants (Fig. 3). In addition, TbSRPPs appear not to contribute to the surface charge and subsequently to the electrostatic repulsion of rubber particles since both, rubber particles from TbSRPP-RNAi transgenic and wild-type plants showed a nearly identical ζ-potential (Fig. 4A). This is in consistence with results obtained for *H. brasiliensis* rubber particles, whose negative surface charge is mainly derived from adsorbed long chain fatty acids and only little from proteins [32].

Depletion of the rubber particle surface charge by suspending rubber particles in a buffer with a pH-value close to their isoelectric point led to an increase of their z-average diameter, indicating the formation of aggregates (Fig. 4B). This suggests that electrostatic repulsion contributes to a great extent to the colloidal stability of *T. brevicorniculatum* rubber particles. Nevertheless, even at pH 7.2 the rubber particle ζ-potential of approximately −18 mV remains insufficient for electrostatic force to solely provide particle stabilization, which requires a ζ-potential of at least ±30 mV [18], [33]. Therefore, the colloidal stability of *T. brevicorniculatum* rubber particles is not exclusively mediated by electrostatic repulsion. This is strongly supported by the fact that the depletion of TbSRPPs, which have no impact on the rubber particle surface charge, led to a significant increase of the z-average diameter of rubber particles from TbSRPP-RNAi transgenic plants compared to wild-type (Fig. 4A, B). Microscopic analysis of purified rubber particles using CLSM and TEM confirmed that even at pH 7.2, where the surface charge of the rubber particles is considerably high, rubber particles from TbSRPP-RNAi transgenic plants aggregated intensely, while wild-type particles remained solitary (Fig. 4A, C–H). Therefore, we suppose that in addition to the electrostatic repulsion of rubber particles (most probably provided by their membrane lipids) TbSRPPs contribute to their stabilization by steric hindrance. This is supported by the fact that at low surface charges rubber particles from TbSRPP*-*RNAi transgenic plants showed an even more intensive aggregation compared to wild-type particles, suggesting an additive effect of electrostatic repulsion and steric repulsion mediated by TbSRPPs as also considered for other colloidal systems (Fig. 4A, B) [19]–[22]. Accordingly, the monolayer-membrane surrounded seed oil bodies are sterically stabilized by oleosin, their major surface protein [34], [35]. The assumption that proteins on the surface of rubber particles contribute to their stability is also supported by the fact that *H. brasiliensis* rubber particles become unstable when treated with proteolytic enzymes [36].

In summary, our data obtained by photon correlation spectroscopy of rubber particles suggest that TbSRPPs are involved in the stabilization of the rubber particle membrane. As a consequence, TbSRPPs-depleted rubber particles may not provide a suitable environment for the CPT and subsequently efficient IPP-polymerization. To gain deeper insight into the functional role of individual SRPPs in rubber biosynthesis, it will be necessary to generate transcript specific knock-down plants and to analyze their respective effect on rubber biosynthesis and rubber particle biogenesis as well as rubber particle architecture and stability.

## Materials and Methods

### Plant material and cultivation conditions

*T. brevicorniculatum* plants and seeds were obtained from the Botanical Gardens Karlsruhe (Germany) and cultivated in a greenhouse at 18°C and a 16 h photoperiod with 20 kilolux light intensity.

### Preparation of *T. brevicorniculatum* rubber particles

Latex from *T. brevicorniculatum* was harvested as described previously [8]. The latex suspension was separated into three phases by centrifugation at 4°C for 5 min and 8,000×g. The rubber phase and the soluble phase were transferred to a new tube and centrifuged at 4°C for 15 min at 10,000xg. The soluble phase was isolated using a syringe and afterwards the remaining rubber particle suspension, if not stated otherwise, was diluted with an appropriate volume rubber extraction buffer (REB) [8]. All experiments described were carried out with freshly prepared rubber particles.

### Two-dimensional gel electrophoresis of rubber particle proteins

Fifty microliter of rubber particle suspension was used for protein extraction using the ZOOM® 2D Protein Solubilizer 1 according to the manufacturer's recommendations (Invitrogen, Karlsruhe, Germany) with the following modification: Before rehydration, acetone precipitation was carried out to concentrate proteins. After solubilising proteins in rehydration buffer for at least 4 h, 50 µg protein (determined according to Bradford) [37] was loaded on pH 3–10 7 cm or pH 3–6 7 cm ReadyStrip^TM^ IPG strips (Biorad, Munich, Germany). The strips were rehydrated overnight and isoelectric focussing was carried out at 20°C and a maximum current of 50 µA using the following program: 300 V for 30 min, 1000 V for 30 min, 5000 V until 12 kVh were reached. Proteins were either stained with colloidal Coomassie Brilliant Blue (Fermentas, St. Leon-Rot, Germany) or transferred to nitrocellulose membranes, which were stained with 0.1% Ponceau-S in 5% acetic acid to confirm equal loading of proteins. Membranes were incubated with the anti-TbSRPP antibody (1∶250) for 1 h and a secondary mouse anti-rabbit IgG conjugated to horse radish peroxidase according to manufacturer's recommendation (Sigma, Schnelldorf, Germany). TbSRPPs were visualized on X-ray films by chemiluminescence detection.

### Protein identification by mass spectrometrical analysis

Protein bands were excised from gels and subjected to in-gel trypsin digestion following the descriptions in Shevchenko *et* *al*. [38]. Subsequent LC-MS/MS was accomplished using an Ultimate 3000 system (Dionex, Sunnyvale, CA, USA) for Nano-LC and a LTQ Orbitrap XL mass spectrometer (Thermo, Bremen, Germany) [39]. The five most abundant precursor ions in the first MS were used for MS/MS fragmentations. MS/MS data was searched against a database created from all protein data available at the public NCBI non-redundant (nr) database (http://www.ncbi.nlm.nih.gov/, October 2009 status) using the OMSSA software (version 2.1.4) [40]. Mass tolerances were set to 0.02 Da for the precursor ions and to 0.5 Da for the product ions. An estimation of true protein identification hits was achieved by searching a concatenated database of target and decoy peptide mass information and defining an adaptive E-value threshold for all hits originating from one sample in such a way that the estimated false positive rate was ≤1% and by a mass accuracy requirement of 5 ppm.

### Construction of expression vector for heterologous expression of TbSRPP3 and generation of antibodies against TbSRPPs

The full length cDNA encoding *TbSRPP3* was amplified using the primer combination srpp3-EcoRI and srpp3-rev_NotI (Tab. S1). The cDNA was subsequently inserted into the pCRII-TOPO® vector (Invitrogen, Karlsruhe, Germany), sequenced and then transferred into the expression vector pGEX4-T1, using the EcoRI and NotI restriction sites (GE Healthcare Europe GmbH, Freiburg, Germany). This allowed the expression of TbSRPP3 in C-terminal fusion to glutathione-S-transferase (GST) for heterologous expression in *Escherichia coli* BL21-cells and subsequent antibody generation. The *TbSRPP3* clone in pGEX4-T1 was expressed in 300-ml cultures of *E. coli* strain BL21, induced with 1 mM isopropyl-beta-D-thiogalactopyranoside (IPTG). The protein band containing heterologously expressed TbSRPP3 was excised from the gel, the eluted protein was sequenced by MALDI-MS and send to EUROGENTEC (Seraing, Belgium) for antibody production. The pre-immune and antibody sera were tested by western blot analysis against recombinant TbSRPP1-5 proteins.

### Total RNA extraction from *T. brevicorniculatum* latex and cDNA synthesis

Extraction of total RNA from *T. brevicorniculatum* latex and cDNA synthesis was carried out as previously described [27], whereas random hexamer primer were used to initiate the reverse transcription reaction (Fermentas, St. Leon-Rot, Germany).

### Quantitative real time PCR experiments

Quantitative real time PCR was carried out using an iCycler realtime PCR system (Biorad, Munich, Germany). 7.5 µl of serial cDNA dilutions was added to 12.5 µl IQ^TM^SYBRGreen Supermix and 5 µl Primermix (50 pmol each). The primers used for the amplification of the *TbSRPP* transcripts are listed in table S2. The transcript of the housekeeping gene *TbACTIN* was used as a reference (Tab. S2). Melting curves were analyzed to exclude false amplifications. After determination of the C_t_-values, the relative expression of the genes was calculated using the REST-MCS software [41].

### Cloning of *TbSRPP3*-RNAi construct

For cloning of the *TbSRPP3*-RNAi construct, a 363 bp-fragment specific for all *TbSRPP* genes was obtained from *TbSRPP3* cDNA (coordinates 270–632 of cDNA) by PCR using the primers TbSRPP3 RNAi KpnI fw and TbSRPP3 RNAi XhoI rev (Tab. S2). To avoid off-target suppression of genes, we validated that the chosen cDNA fragment gave no hits for other than TbSRPP1–5 homologs in the public available sequences and especially in EST datasets for the closely related plant species *T. koksaghyz* and *T. officinale* using the Basic Local Alignment Search Tool (BLAST; http://www.ncbi.nlm.nih.gov). The cDNA fragment was inserted into the KpnI and XhoI sites of the Gateway® pENTR^TM^4 and transferred into the pFGC5941 (www.chromDB.org) using the Gateway®LR Clonase enzyme mix (Invitrogen, Karlsruhe, Germany). The integrity of the construct was verified by sequencing.

### *Agrobacterium*-mediated transformation of *T. brevicorniculatum*

*Agrobacterium*-mediated transformation was done as previously described [29] using the *Agrobacterium tumefaciens* strain EHA105 that has been transformed with the binary plasmid.

### Determination of dry rubber content

To determine the dry rubber content in exuding latex, roots of *T. brevicorniculatum* plants were dissected with a razor blade and 20 µl of the expelling latex was transferred to 100 µl REB supplemented with 5 mM DTT. The samples were centrifuged at 4°C and 12000×g and overlaid with 100 µl methanol to coagulate rubber particles. The rubber coagulum was air-dried for 24 h and weighted. To determine the dry rubber content of whole roots, the freeze-dried entire roots were ground into a fine powder using a coffee grinder. For extraction, 200 mg root powder and 4 ml toluene were mixed thoroughly and incubated at 70°C for 18 h. The organic phase was transferred to a new glass vial and toluene was evaporated. The remaining material was dissolved in 300 µl toluene for 30 min and then 600 µl methanol was added to precipitate the rubber. After 30 min the samples were centrifuged at RT and 16,000×g for 2 min. The precipitated rubber was washed with water and acetone by vigorous shaking for 30 min at RT to remove proteins and resins. Subsequently the rubber samples were dried and weighted.

### Size exclusion chromatography of *T. brevicorniculatum* rubber

Toluene was added to air-dried rubber samples to a final concentration of 0.75 mg ml^−1^ and dissolved at 60°C for 16 h. The samples were filtered with Cameo 3 N syringe (Nylon, 0.22 micron) filters (Roth, Karlsruhe, Germany) and transferred to new glass vials. Size exclusion chromatography (SEC)-analysis was carried out using a SECcurity GPC system with a column combination including a PSS SDV 20 µ 8.0×50 mm precolumn, a PSS SDV Linear XL 20 µ 8×300 nm analytical column and a PSS SDV 100 Å 20 µ 8×300 mm analytical column (Polymer Standards Service, Mainz, Germany).

### [1-^14^C] IPP incorporation assay

The [1-^14^C] IPP incorporation rate of crude latex was determined as described previously [10].

### Determination of rubber particle ζ-potential and z-average diameter

20 µl of rubber particle suspension was diluted in 1180 µl modified REB (350 mM Sorbitol, 5 mM MgCl, 50 mM NaCl, 40 mM NaH_2_PO_4_ titrated with 20 mM Citrate to the desired pH) to reach a minimal derived count rate of 250 kcps. One milliliter of the suspension was injected into a Malvern Clear Zeta Potential Cell. The Derjaguin-Landau-Verwey-Overbeek theory allows the quantification of the electrostatic stability of lyophobic colloids by measuring their ζ-potential (Verwey, 1947). The electrophoretic mobility of rubber particles was measured by Laser Doppler Velocimetry and was converted to ζ-potential by the Dispersion Technology Software (DTS) using Henrýs equation and the Smoluchowski approximation. The intensity weighted mean hydrodynamic diameter (z-average diameter) was measured by dynamic light scattering. The ζ-potential and z-average diameter of rubber particles at different pH-values were determined using a Zetasizer Nano ZS and DTS (Malvern Instruments Ltd., Worcestershire, United Kingdom).

### Scanning electron microscopy

Rubber particles were fixed with 2.5% glutaraldehyde in 0.05 M sodium cacodylate buffer for 1 hour at room temperature. After 3 times of washing with 0.05 M sodium cacodylate buffer pH 7.3 (at 5,000×g), they were postfixed with 1% osmium tetroxide for 1 hour. Finally the particles were washed 3 times with 0.05 M sodium cacodylate buffer and subsequently 3 times with PBS (pH 7.4), each for 5 minutes.

For immunogold labeling, 10 µl aliquots of the suspensions were placed on a freshly glow-discharged carbon-layer (thickness ∼10 nm) onto a Pioloform film supported by a Cu-mesh grid, let sediment for 5 minutes, and washed with PBS (pH 7.4) for 10 minutes. The grids were then incubated in PBS buffer containing 5% bovine serum albumin (BSA) (pH 7.4) for 15 minutes, followed by 3 times washing with PBS (pH 7.4) each for 5 minutes. After this, the grids were incubated for 3 hours either with the primary anti-TbSRPP antibody (1∶5 in PBS, pH 7.4) or with the pre-immunserum (1∶5 in PBS, pH 7.4) as a negative control. Subsequently, grids were washed 5 times for 5 minutes with PBS (pH 7.7) and then 5 minutes with Tris-BSA-buffer (pH 8.2). After this the samples were incubated with a secondary goat-anti rabbit IgG antibody coupled to 10 nm colloidal gold particles (Sigma-Aldrich, Schnelldorf, Germany) diluted 1∶20 in Tris-BSA-buffer (pH 8.2) for 2 hours. Finally, samples were washed with Tris-BSA-buffer (pH 8.2) 6 times for 5 minutes and once with distilled water for 5 minutes. After air drying, the sample was covered by 2 nm platinum/carbon. Images were recorded by a field emission SEM S-5000 (Hitachi, Japan) at electron energy of 20 kV; both, secondary and backscattered electron signals were recorded.

### Transmission electron microscopy

TEM of ultra-thin root cross sections was carried out as described previously [10].

### Confocal laser scanning microscopy

Fifty microliters of freshly prepared rubber particles were supplemented with 1 µl nile red (15 µM solubilised in DMSO) and fluorescence was visualized by CLSM (excitation 514 nm, emission 509–620 nm, Leica DMIRE2, Wetzlar, Germany).

## Supporting Information

Figure S1 **Anti-TbSRPP antibody detects heterologously expressed TbSRPP1–5. A.** Protein extracts from pellet and supernatant from *E. coli* cultures heterologously expressing TbSRPP1–5 proteins were separated by SDS-PAGE and subsequently stained with Coomassie Brilliant Blue. **B.** Protein extracts from pellet and supernatant from *E. coli* cultures heterologously expressing TbSRPP1–5 proteins were separated by SDS-PAGE and subsequently transferred to a nitrocellulose membrane. TbSRPP1–5 were detected using the anti-TbSRPP antibody and a secondary antibody coupled with alkaline phosphatase (1, TbSRPP1; 2, TbSRPP2; 3, TbSRPP3; 4, TbSRPP4; 5, TbSRPP5; P, pellet; S, supernatant).
(TIF)

Table S1 **Identification of TbSRPP2, TbSRPP3, TbSRPP4, and TbSRPP5 on** ***T. brevicorniculatum*** **rubber particles by mass spectrometric analysis.**
(DOC)

Table S2 **Oligonucleotide sequences and respective assignments.**
(DOC)

Text S1 **Materials and Methods section regarding experiments performed for Figure** **S1.**
(DOC)
