# Supplementary figures and images for: Down-Regulation of Small Rubber Particle Protein Expression Affects Integrity of Rubber Particles and Rubber Content in Taraxacum brevicorniculatum
Source: PLoS One. 2012 Jul 23;7(7):e41874. doi: 10.1371/journal.pone.0041874 (PMC3402443; doi:10.1371/journal.pone.0041874)

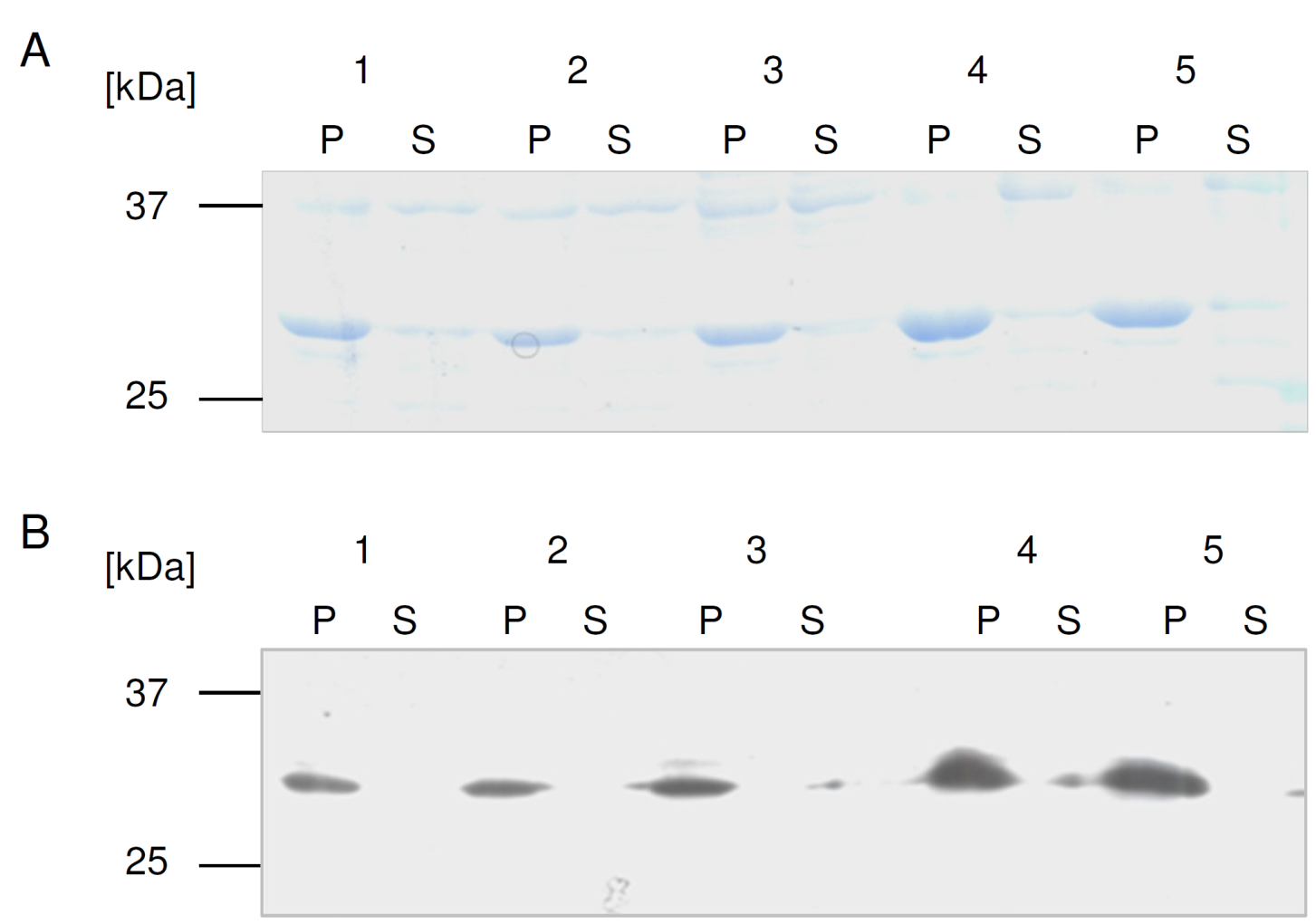

Supplement: Figure S1 — Anti-TbSRPP antibody detects heterologously expressed TbSRPP1–5. A. Protein extracts from pellet and supernatant from E. coli cultures heterologously expressing TbSRPP1–5 proteins were separated by SDS-PAGE and subsequently stained with Coomassie Brilliant Blue. B. Protein extracts from pellet and supernatant from E. coli cultures heterologously expressing TbSRPP1–5 proteins were separated by SDS-PAGE and subsequently transferred to a nitrocellulose membrane. TbSRPP1–5 were detected using the anti-TbSRPP antibody and a secondary antibody coupled with alkaline phosphatase (1, TbSRPP1; 2, TbSRPP2; 3, TbSRPP3; 4, TbSRPP4; 5, TbSRPP5; P, pellet; S, supernatant). (TIF) [file pone.0041874.s001.tif]
