# Supplementary material for: Down-Regulation of Small Rubber Particle Protein Expression Affects Integrity of Rubber Particles and Rubber Content in Taraxacum brevicorniculatum
Source: PLoS One. 2012 Jul 23;7(7):e41874. doi: 10.1371/journal.pone.0041874 (PMC3402443; doi:10.1371/journal.pone.0041874)
Supplement: Table S1 — Identification of TbSRPP2, TbSRPP3, TbSRPP4, and TbSRPP5 on T. brevicorniculatum rubber particles by mass spectrometric analysis. (DOC) [file pone.0041874.s002.doc]

**Table S1 Identification of TbSRPP2, TbSRPP3, TbSRPP4, and TbSRPP5 on *T. brevicorniculatum* rubber particles by mass spectrometric analysis**

| protein name | peptide count | aminoacid coordinates | peptide sequence | coverage [%]^a^ |
| --- | --- | --- | --- | --- |
| TbSRPP2 | 1 | 191-202 | VSSYLPLVPTER | 6 |
|  |  |  |  |  |
| TbSRPP3 | 18 | 125-129 | ELLVK | 62 |
|  |  | 22-31 | HLDFVEDGVK |  |
|  |  | 88-97 | FDEVMPPAVK |  |
|  |  | 105-115 | SLSTNVASEVK |  |
|  |  | 169-179 | YNETVQQSAEK |  |
|  |  | 183-194 | VSSYVPLVPTDK |  |
|  |  | 157-168 | AVAPTASYYSEK |  |
|  |  | 87-97 | KFDEVMPPAVK |  |
|  |  | 20-31 | LKHLDFVEDGVK |  |
|  |  | 144-156 | TLNYVPYVTTFAK |  |
|  |  | 130-143 | IEPLAEEYASSAWK |  |
|  |  | 105-124 | SLSTNVASEVKNNGVLGTAK |  |
|  |  | 217-238 | EVPGGGEGGEAAAGGDEIVEET |  |
|  |  | 157-179 | AVAPTASYYSEKYNETVQQSAEK |  |
|  |  | 48-72 | SGPLKPHVETLESTIKPVVGPAYDK |  |
|  |  | 215-238 | DKEVPGGGEGGEAAAGGDEIVEET |  |
|  |  | 46-72 | DKSGPLKPHVETLESTIKPVVGPAYDK |  |
|  |  |  |  |  |
| TbSRPP4 | 25 | 121-125 | ELLIK | 89 |
|  |  | 140-149 | TISDLPLVAK |  |
|  |  | 112-120 | NVGVVETVK |  |
|  |  | 101-111 | SLSTTVVSDVK |  |
|  |  | 179-190 | VSSYLPLVPTDK |  |
|  |  | 84-96 | VAQLDSVLPPSVK |  |
|  |  | 112-125 | NVGVVETVKELLIK |  |
|  |  | 150-164 | LAAAIAPLATYITEK |  |
|  |  | 165-178 | YNGTVQQTADEGYK |  |
|  |  | 126-139 | MEPTVEDYASSAWK |  |
|  |  | 64-78 | TYGGPAYDTLNGVLK |  |
|  |  | 84-100 | VAQLDSVLPPSVKETAK |  |
|  |  | 46-63 | ENAGTLKPGVETIETTVK |  |
|  |  | 201-218 | EDEEVPGGLGGEEATEVP |  |
|  |  | 22-39 | HLGFVEEGIQQVAGYASK |  |
|  |  | 79-96 | FADNKVAQLDSVLPPSVK |  |
|  |  | 2-21 | ADVAPVTDELEVQSEKETLK |  |
|  |  | 101-120 | SLSTTVVSDVKNVGVVETVK |  |
|  |  | 79-100 | FADNKVAQLDSVLPPSVKETAK |  |
|  |  | 40-63 | VYDYAKENAGTLKPGVETIETTVK |  |
|  |  | 194-218 | IFSTSTKEDEEVPGGLGGEEATEVP |  |
|  |  | 101-125 | SLSTTVVSDVKNVGVVETVKELLIK |  |
|  |  | 165-190 | YNGTVQQTADEGYKVSSYLPLVPTDK |  |
|  |  | 150-178 | LAAAIAPLATYITEKYNGTVQQTADEGYK |  |
|  |  | 150-190 | LAAAIAPLATYITEKYNGTVQQTADEGYKVSSYLPLVPTDK |  |
|  |  |  |  |  |
| TbSRPP5 | 24 | 73-82 | YHDVPVVVLK | 93 |
|  |  | 117-128 | EFLEKIDPVAEK |  |
|  |  | 149-160 | ALTPSAALVTEK |  |
|  |  | 167-179 | ENASFLPLVPTDK |  |
|  |  | 109-121 | VGVVETAKEFLEK |  |
|  |  | 48-63 | SGPLKPGVETIETTLK |  |
|  |  | 167-182 | ENASFLPLVPTDKIAR |  |
|  |  | 88-104 | VDESVTQIDGVLPPIVK |  |
|  |  | 22-39 | YLEFVEEAITQAIDYASK |  |
|  |  | 46-63 | EKSGPLKPGVETIETTLK |  |
|  |  | 87-104 | KVDESVTQIDGVLPPIVK |  |
|  |  | 149-166 | ALTPSAALVTEKYNQTVK |  |
|  |  | 161-179 | YNQTVKENASFLPLVPTDK |  |
|  |  | 64-82 | TVVGPAYEKYHDVPVVVLK |  |
|  |  | 129-148 | YASSAWSTVNQLPLVASTVK |  |
|  |  | 88-108 | VDESVTQIDGVLPPIVKDATK |  |
|  |  | 2-21 | ADAASVTDEPQVQTEGEKLK |  |
|  |  | 87-108 | KVDESVTQIDGVLPPIVKDATK |  |
|  |  | 122-148 | IDPVAEKYASSAWSTVNQLPLVASTVK |  |
|  |  | 190-217 | DAEKPEPAVVPGGEEEAAEEVAGGGGAE |  |
|  |  | 149-179 | ALTPSAALVTEKYNQTVKENASFLPLVPTDK |  |
|  |  | 183-217 | VFSIPEKDAEKPEPAVVPGGEEEAAEEVAGGGGAE |  |

**^a^**sequence coverage was rounded to the nearest whole percentage.
