# Supplementary material for: Down-Regulation of Small Rubber Particle Protein Expression Affects Integrity of Rubber Particles and Rubber Content in Taraxacum brevicorniculatum
Source: PLoS One. 2012 Jul 23;7(7):e41874. doi: 10.1371/journal.pone.0041874 (PMC3402443; doi:10.1371/journal.pone.0041874)
Supplement: Table S2 — Oligonucleotide sequences and respective assignments. (DOC) [file pone.0041874.s003.doc]

**Table S2 Oligonucleotide sequences and respective assignments**

| Primer | Sequence 5´-3´(restriction sites are underlined) | ***Assignment*** |
| --- | --- | --- |
| srpp3-EcoRI | TTT GAA TTC ATG ACC GAC GCT GCT TCTG | Heterologous expression of TbSRPP3 |
| srpp3-rev_NotI | AAA GCG GCC GCT CATG TTT CCT CCA CAA TCT CAT CC |
| TbSRPP1 qRT fw | GCC GAT AAC GCT GTT CCT GTT | Quantitative real time PCR |
| TbSRPP1 qRT rev | GGC TTG CTT TGC TGC TTC TTG |
| TbSRPP2 qRT fw | CGA TGC TCC TGT TAC TAA TCA ACC | Quantitative real time PCR |
| TbSRPP2 qRT rev | CAT GAA TAG CCG CCA CTT GAA CAA A |
| TbSRPP3 qRT fw | CGA CGC TGC TTC TGT TAC TGA | Quantitative real time PCR |
| TbSRPP3 qRT rev | ATA ACC AAC TGC TTG CTT TAC TCC |
| TbSRPP4 qRT fw | CCG ATG TTG CAC CTG TTA CC | Quantitative real time PCR |
| TbSRPP4 qRT rev | CTT GCT GTA TTC CCT CTT CTA CG |
| TbSRPP5 qRT fw | GAT GAA CCA CAG GTC CAG AC | Quantitative real time PCR |
| TbSRPP5 qRT rev | CCT TAG CAA ACT CAT ATA CTT TTG ATG C |
| Tbactin qRT fw | CGACCTCATACCTATTCCCAC | Quantitative real time PCR |
| Tbactin qRT fw | CAGCCTTCACCATTCCAGTTC |
| TbSRPP3 RNAi KpnI fw | AAA GGT ACC GGT AAT GCC TCC TGC GGT G | Cloning of TbSRPP3 RNAi construct |
| TbSRPP3 RNAi XhoI rev | AAA CTC GAG CCT CCG GGA CCC GCT GAT TC |
