# Supplementary material for: Down-Regulation of Small Rubber Particle Protein Expression Affects Integrity of Rubber Particles and Rubber Content in Taraxacum brevicorniculatum
Source: PLoS One. 2012 Jul 23;7(7):e41874. doi: 10.1371/journal.pone.0041874 (PMC3402443; doi:10.1371/journal.pone.0041874)
Supplement: Text S1 — Materials and Methods section regarding experiments performed for Figure S1. (DOC) [file pone.0041874.s004.doc]

**Materials and Methods section regarding experiments performed for Figure S1**

For heterologous expression of the TbSRPPs the full length cDNAs of the genes were amplified and cloned into suitable expression vectors. For cloning of *TbSRPP1*, the full length cDNA was amplified using the primers TbSRPP1 BspHI fw (5´- ATA TCA TGA ATG GCC GAT AAC GCT GTT C-3´) and TbSRPP1NotI rev (5´- AAA GCG GCC GCT CAA TGT GCC ACG ACA GC-3´) and (using the restriction sites NcoI and NotI of the plasmid) ligated into pET-Duet-1. For cloning of TbSRPP2, the cDNA was amplified using the primers TbSRPP2 BamHI fw (5´- AAA GGA TCC ATG GCC GAA AAC GAT GCT C-3´) and TbSRPP2 XhoI rev (5´- ATA CTC GAG TCA AAC AGG GTT GAA GAC C-3´) and also ligated into pET-Duet 1. For cloning of TbSRPP3, the cDNA was amplified using the primers TbSRPP3 BamHI fw (5´- TTT GGA TCC ATG ACC GAC GCT GCT TCT-3´) and TbSRPP3 XhoI rev (5´- ATA CTC GAG TCA TGT TTC CTC CAC AAT-3´) and ligated into the pCOLA-Duet 1. For cloning of TbSRPP4, the cDNA was amplified using the primers TbSRPP4 BamHI fw (5´- TTT GGA TCC ATG GCC GAT GTT GCA CCT G-3´) and TbSRPP4 XhoI rev (5´- ATA CTC GAG TTA AGG AAC CTC CGT AGC-3´) and ligated into the pACYC-Duet 1. For cloning of TbSRPP5, the cDNA was amplified using the primers TbSRPP5 BamHI fw (5´- TTT GGA TCC ATG GCC GAC GCT GCT TCT G-3´) and TbSRPP5 XhoI rev (5´- TTT CTC GAG TTA CTC CGC TCC ACC ACC-3´) and ligated into the pCDF-Duet 1. After verification of the integrity of the constructs by sequencing, TbSRPP1-5 were expressed in 40 ml cultures of the *E. coli* strain Tuner^TM^ induced with 1 mM IPTG. After harvest, the cells were resuspended in 500 µl PBS and subsequently sonicated. The proteins were separated by SDS-PAGE and either directly stained with Coomassie Brilliant Blue or transferred to a nitrocellulose membrane for subsequent western blot analysis. Proteins were detected using the primary anti-TbSRPP antibody and a secondary goat-anti-rabbit IgG conjugated to alkaline phosphatase according to manufacturer’s recommendation (Sigma, Schnelldorf, Germany). Alkaline phosphatase activity was visualized using SIGMA *FAST™* BCIP/NBT (5-Bromo-4-chloro-3-indolyl phosphate/Nitro blue tetrazolium) Tablets according to the manufacturer’s recommendation (Sigma, Schnelldorf, Germany).
